# Riding the crest of the wave: parallels between the neural crest and cancer in epithelial-to-mesenchymal transition and migration

**DOI:** 10.1002/wsbm.1224

**Published:** 2013-04-10

**Authors:** Davalyn R Powell, Alex J Blasky, Steven G Britt, Kristin B Artinger

**Affiliations:** 1 Graduate Program in Cell Biology, Stem Cells and Development, School of Medicine, University of Colorado Anschutz Medical CampusAurora, CO, USA; 2 Department of Craniofacial Biology, School of Dental Medicine, University of Colorado Anschutz Medical CampusAurora, CO, USA; 3 Department of Pediatrics, School of Medicine, University of Colorado Anschutz Medical CampusAurora, CO, USA; 4 Department of Cell and Developmental Biology, School of Medicine, University of Colorado Anschutz Medical CampusAurora, CO, USA; 5 Department of Ophthalmology, School of Medicine, University of Colorado Anschutz Medical CampusAurora, CO, USA

## Abstract

The neural crest (NC) is first induced as an epithelial population of cells at the neural plate border requiring complex signaling between bone morphogenetic protein, Wnt, and fibroblast growth factors to differentiate the neural and NC fate from the epidermis. Remarkably, following induction, these cells undergo an epithelial-to-mesenchymal transition (EMT), delaminate from the neural tube, and migrate through various tissue types and microenvironments before reaching their final destination where they undergo terminal differentiation. This process is mirrored in cancer metastasis, where a primary tumor will undergo an EMT before migrating and invading other cell populations to create a secondary tumor site. In recent years, as our understanding of NC EMT and migration has deepened, important new insights into tumorigenesis and metastasis have also been achieved. These discoveries have been driven by the observation that many cancers misregulate developmental genes to reacquire proliferative and migratory states. In this review, we examine how the NC provides an excellent model for studying EMT and migration. These data are discussed from the perspective of the gene regulatory networks that control both NC and cancer cell EMT and migration. Deciphering these processes in a comparative manner will expand our knowledge of the underlying etiology and pathogenesis of cancer and promote the development of novel targeted therapeutic strategies for cancer patients. © 2013 Wiley Periodicals, Inc.

## INTRODUCTION

**FIGURE 1:**
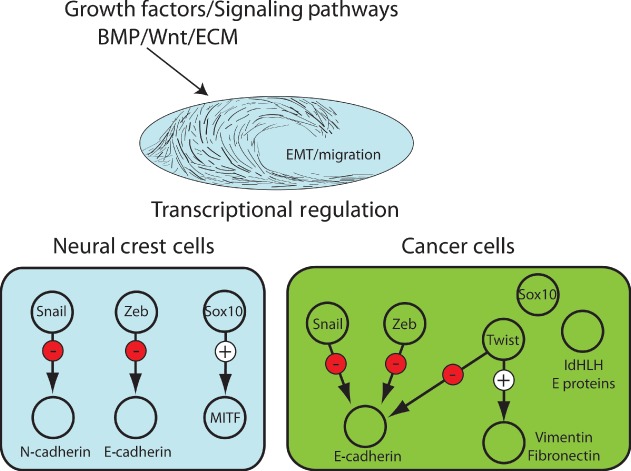
Comparison of transcriptional regulation in neural crest (NC) cells and cancer cells. The diagram depicts the role of growth factors and their signaling pathways in initiating epithelial-to-mesenchymal transition (EMT) and migration in both NC cells and cancer cells. The actions of the specific transcription factors discussed in the text are indicated.

**FIGURE 2:**
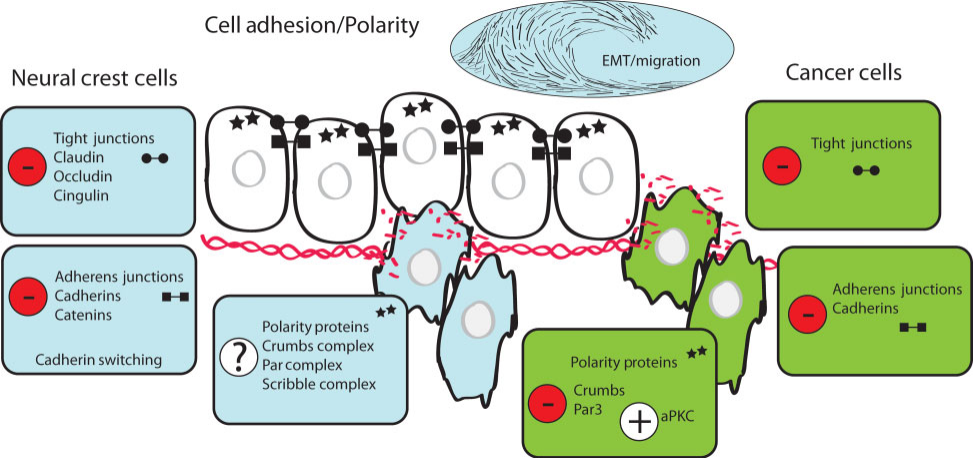
Comparison of cell adhesion and polarity changes in neural crest (NC) cells and cancer cells. The diagram depicts alterations in tight junction components (circle barbells), adherens junction components (square barbells), and polarity proteins (stars) during epithelial-to-mesenchymal transition (EMT) and migration in NC cells (light blue) and cancer cells (green) as they differentiate and migrate away from normal epithelial cells (white). Passage of cells through the basement membrane and disruption of the extracellular matrix (ECM; red intertwined lines and red broken fragments) is indicated.

**FIGURE 3:**
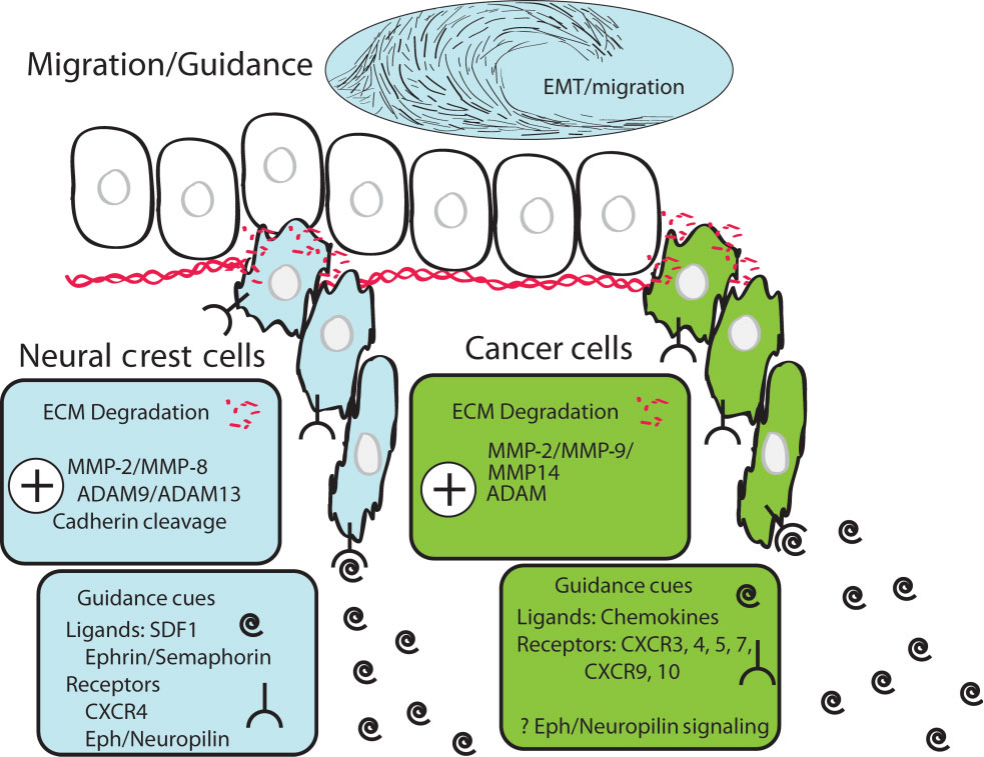
Comparison of migration and guidance in neural crest (NC) cells and cancer cells. The diagram depicts degradation of the extracellular matrix (ECM; red intertwined lines and red broken fragments) and the role of guidance cues (ligands shown as spirals and receptors shown as 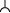) during EMT and migration in NC cells (light blue) and cancer cells (green).

The neural crest (NC) is a population of transient, multipotent cells that are specified at the border of the neural plate between the neural and non-neural ectoderm in vertebrate embryos. These cells undergo an epithelial-to-mesenchymal transition (EMT), delaminate, and migrate away from the neural tube to populate various tissues and contribute multiple cell fates to the developing embryo, including pigment cells, neurons and glia of the peripheral nervous system, and craniofacial cartilage.^1^,^2^ The genes that regulate these developmental processes have been extensively studied in many model systems, including *Xenopus*, zebrafish, chick, and mouse, and are highly conserved between these vertebrate species. The process of EMT involves downregulation of characteristic epithelial genes such as the adhesion genes E-cadherin, Claudins, and Occludins and the upregulation of mesenchymal markers such as fibronectin, vitronectin, and vimentin (Figures 1 and 2). After EMT, NC cell migration involves complex interactions between the cells and the environment in which they migrate including positive responses to attractive signals such as chemokines, as well as avoidance of repulsive signals such as ephrins and semaphorins (Figure 3). In combination, these signals direct NC cells along restricted migratory paths. In addition, NC cells use cell autonomous activation of matrix metalloproteinases (MMPs) and ADAMs (a disintegrin and metalloproteinase; adamlysins) to break down the extracellular matrix (ECM), including proteins such as fibronectin, and to facilitate migration to their final destinations.

The processes described for NC development share many characteristics with the progression of cancer. For example, primary epithelial tumors display EMT characteristics similar to those displayed during NC EMT before they delaminate and begin to metastasize. Key features of cancer progression can involve a loss of junctional proteins, disruption of the basement membrane, and upregulation of mechanisms to escape cell death. While NC cell EMT and migration are tightly regulated and conserved processes, a key feature of cancer cell movement is a misregulation of these same processes. This includes the usurping of developmental programs including those that direct cell polarity, adhesion, and cell morphology. For instance, during cancer EMT, abnormal upregulation of key developmental transcriptional regulators such as the Snail, Twist, and ZEB transcription factors occurs.^3^–^6^ Similar to NC cells, as cancer cells migrate during metastasis, they can upregulate proteins such as MMPs to break down the ECM and invade tissues to form metastatic lesions. Additionally, recent studies suggest that, like NC cells, migrating tumor cells respond to chemoattractive signals that promote migration to their secondary invasion site. The parallels between the genes and proteins involved in EMT and migration in both NC development and cancer progression make NC cells an excellent model for investigating the genetic regulation of cancer progression and metastasis (summarized in Table 1). Several recent reviews have documented the similarities between NC development and cancer migration.^7^–^9^ Here, we directly compare at a systems level NC and cancer cell EMT and migration, and demonstrate how this information provides a unique perspective on cancer cell migration and metastasis.

**TABLE 1:** Genes with Roles in Both Neural Crest Development and Cancer Progression

Gene	Role in Neural Crest Development	Role in Cancer Progression
Transcription factors		
Snail/Slug	Induction of EMT^10^–^13^	Induction of EMT and metastasis^3^,^14^–^19^
Zeb	Induction of EMT and NC migration^20^–^24^	Induction of EMT and metastasis^3^,^25^
Sox10	NC differentiation (melanocytes) and survival^26^–^30^	Melanoma formation^31^
Twist	Induction of EMT and NC migration, differentiation^32^,^33^	Induction of EMT and metastasis^34^–^36^
Adhesion and polarity		
Claudins and Occludins	Downregulated for migration^37^–^40^	Downregulation correlates with migration^41^
E-cadherin	Downregulated during NC specification^42^–^44^	Downregulation correlates with migration^45^
N-cadherin	Downregulated for NC delamination and migration^10^,^42^,^46^–^48^	Downregulation correlates with migration^49^,^50^
Crumbs complex	Polarity of NC cells^51^	Downregulation correlates with EMT^52^–^55^
Par complex	Polarity of NC cells^51^	Misregulation correlates with EMT^56^–^59^
Migration		
MMPs and ADAMs	Degradation of ECM for NC migration^60^–^65^	Degradation of ECM for metastasis^66^–^76^
SDF1/CXCR4	Directional NC migration^77^–^79^	Metastasis and survival^80^,^81^
Ephrins and semaphorins	Directional NC migration^8^,^82^–^85^	Misregulated in cancer^86^,^87^

ECM, extracellular matrix; EMT, epithelial-to-mesenchymal transition; MMPs, matrix metalloproteinases; NC, neural crest.

## TRANSCRIPTION FACTORS IN NC EMT AND CANCER

**FIGURE 4:**
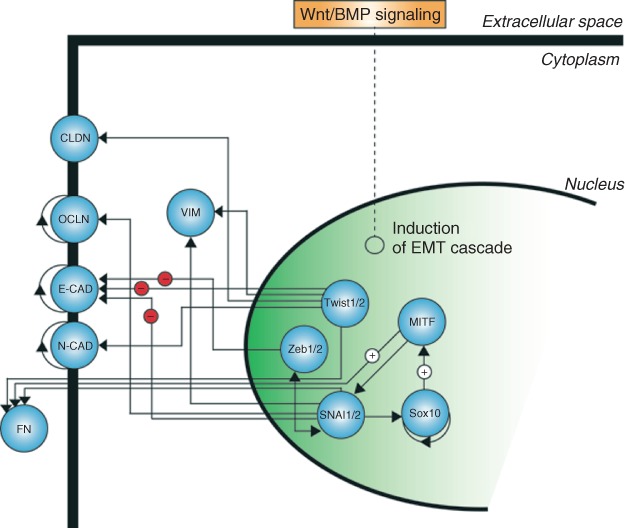
Pathway analysis showing integration between signaling pathways, transcription factors, and adhesion genes. Wnt and BMP act at the top of the hierarchy to initiate the induction of EMT cascade. The transcriptional network, including Twist1/2, Zeb1/2, Snail1/2, Sox10, and Mitf, is active both in neural crest development and cancer. The transcription factors then interact with adhesion genes on the cell surface including N-cadherin (N-cad), E-cadherin (E-cad), Occludins (Ocln), Claudins (Cldn), and extracellular matrix proteins such as fibronectin (FN) and intermediate filament proteins such as vimentin (Vim). (The network was built with Ingenuity Pathway Analysis, Ingenuity Systems, Inc., Redwood City, CA and modified in Adobe Illustrator).

EMT in both the NC and cancer is triggered by various signaling pathways including BMP, Wnt, and signals from the ECM.^4^,^8^ One of the hallmark targets of these signaling pathways are the Snail family of transcription factors including Snail1 and Snail2 (also known as Slug), which are known to have an important role in both NC specification and EMT as well as in cancer progression (Figures 1 and 4). Snail is expressed in the NC prior to EMT in both zebrafish and chick embryos, following initial specification of the NC.^88^,^89^ Snail is thought to be an initial early target of transcription factors that specify the NC or ‘NC specifiers’ such as Foxd3 in zebrafish.^90^ In chick, once Snail transcription factors are expressed in the NC, they initiate EMT by repressing N-cadherin and thereby mediating the switch to a mesenchymal phenotype.^10^ In chick and *Xenopus*, loss of Snail family members results in a failure of EMT, and failure of NC delamination and migration.^11^,^12^ Similarly, in zebrafish, loss of the two Snail transcription factors results in massive embryonic failure of EMT in multiple tissues.^13^ However, in the mouse, the requirement for Snail in NC formation and delamination may not be conserved.^91^ Snail proteins have also been found to be crucial for cancer cell EMT through similar mechanisms. Snail1 has been shown to transcriptionally repress E-cadherin in invasive carcinoma cells and epithelial tumors.^14^,^15^ Furthermore, upregulation of Snail proteins has been shown to correlate with an increased incidence of tumor metastasis, recurrence, and an overall poorer prognosis in human cancers including breast, ovarian, colon, liver, and squamous cell carcinomas, suggesting that Snail is involved in increased EMT and tumor cell migration in human patients.^3^ In addition, inhibition of Snail in tumor cell lines or in orthotopic mouse models can reverse EMT and invasiveness.^16^–^18^ Interestingly, in studies of transformed primary human melanocytes, which form metastatic melanomas in nude mice, the upregulation of Snail2 expression is required for metastatic spread.^19^ Furthermore, in patients with benign nevi that have not undergone malignant transformation, Snail2 is highly expressed and is significantly correlated with the expression of other NC migration-associated genes.^19^ These data suggest that while expression of Snail2 is not sufficient for malignant transformation and malignant spread, Snail2 and other NC developmental genes may play an important role in the biology of malignant melanoma and other invasive cancers.

Another key family of factors that regulate EMT in NC and cancer cells is the Zeb family of transcription factors, containing Zeb1 and Zeb2. Zeb transcription factors are expressed in the NC in *Xenopus* and mouse during early development, as well as in a subset of NC derivatives.^92^ Loss of Zeb factors leads to a defect in NC migration in the mouse embryo and a persistence of E-cadherin after differentiation of the neuroepithelium from the ectoderm and after EMT,^20^,^21^ correlating with the role of Zeb proteins as transcriptional repressors of E-cadherin.^22^ Furthermore, mutations in the human Zeb protein have also been linked to the neurocristopathy Hirschprung's disease, which is characterized by a failure of enteric NC cells to migrate into and populate the gut.^23^,^24^ Zeb factors also repress E-cadherin in tumor progression. Similar to Snail, high expression levels of Zeb1 or Zeb2 correlate with a decrease in E-cadherin expression in a multitude of human cancers including breast, endometrial, colon, uterine, pancreatic, and non-small cell lung cancers.^3^,^25^ This suggests that Zeb factors correlate with increased metastasis and poor prognosis.

The transcription factor Sox10 is also an important activator of NC fate and functions at many stages of NC cell development. The pattern of Sox10 expression in the NC is highly conserved across zebrafish, *Xenopus*, chick, and mice, and it is also expressed in human NC precursors.^26^ Initially expressed at the premigratory stage, Sox10 expression is maintained in most migratory NC progenitors. However, Sox10 functions predominantly in cell differentiation and survival, as NC cells lacking Sox10 form and migrate normally but undergo apoptosis prior to terminal differentiation.^26^,^27^ For example, Sox10 regulates differentiation of the melanocyte lineage through direct transcriptional regulation of the microphthalmia-associated transcription factor (MITF).^28^ Accordingly, mutations in the Sox10 gene disrupt differentiation of the melanocyte lineage, such as in the murine *Dominant megacolon* (*Dom*) and zebrafish *colorless* (*cls*) mutants.^29^,^30^ In addition to a role in NC development, recent work suggests a critical role for Sox10 in the development and persistence of human cancer. In human patients, virtually all congenital nevi and melanomas have upregulated SOX10 expression. Furthermore, in a mouse model of melanoma, loss of one allele of *Sox10* or knockdown with shRNA in human melanoma cells completely abolishes *in vivo* melanoma formation.^31^ These results suggest that targeting of Sox10 expression may suppress the formation of giant congenital nevi and melanomas in human patients.

Additional transcription factors such as the helix-loop-helix (HLH) family including Twist1, E proteins, and Id HLH proteins also have a demonstrated role in EMT. Some of these proteins are known to repress E-cadherin expression, similar to Snail and Zeb, but also may have a role in cell cycle and proliferation control.^93^ Twist1 is required in the developing mouse NC for proper migration and differentiation.^32^,^33^ In cancer, Twist is a repressor of E-cadherin and also activates the expression of several mesenchymal genes such as vimentin and fibronectin.^34^ It is thought that Twist1 induces EMT by activating Snail2.^35^ Moreover, increased Twist expression is associated with later-stage progression of tumors and correlates with increased invasion and metastasis as well as poor survival in human cancer.^36^ Other HLH proteins such as Id proteins have also been shown to be deregulated in a number of human cancers, suggesting that their roles in developmental EMT could be recapitulated in cancer progression.^94^

## CHANGES IN CELLULAR ADHESION AND POLARITY ARE REQUIRED FOR NC AND CANCER EMT

Both NC cell development and cancer metastasis rely on the dynamic reorganization of cellular adhesions during EMT and migration.^95^–^97^ The transition from an epithelial adhesive cellular phenotype to a migratory mesenchymal phenotype is a key feature of NC cell development. As NC cells arise from the neuroepithelium, they exhibit epithelial cell adhesion. Epithelial cell adhesion is maintained through two intercellular adhesion complexes: tight junctions and adherens junctions.

Tight junctions are comprised of families of transmembrane proteins, Claudins and Occludins, which localize to the apical zone in neuroepithelial cells and maintain adhesion with adjacent cells. Increasing evidence implicates the disruption of tight junctions as a critical step during NC cell EMT and migration (Figures 2 and 4). Claudins and Occludins are downregulated in the neural tube prior to NC cell migration,^37^ and Snail, a known transcriptional mediator of EMT, has been shown to directly repress Claudin and Occludin gene expression.^38^,^39^ Furthermore, the downregulation of the tight junction protein Claudin-1 promotes migration of chick cranial NC, whereas overexpression impedes crest migration.^40^ Additionally, inhibition of the tight junction-associated scaffolding protein Cingulin was recently shown to increase the size of the migratory NC cell domain.^98^ In cancer cell biology, disruption of tight junctions is thought to reduce cell adhesion allowing for cancer cell migration, as well as to increase vascular permeability and metastatic spread. Misregulation of tight junction proteins has been observed in a vast range of primary human cancers and model systems, including cancer of the breast, lung, brain and peripheral nerves, skin, oral cavity, endocrine organs, and genitourinary and gastrointestinal tracts.^41^

The disruption of adherens junctions has also been described in both NC cell development and cancer metastasis. Adherens junctions are comprised of several proteins, including classical cadherins and catenins. Classical cadherins form the core of adherens junctions, stabilizing adhesion to neighboring cells through the homophilic interaction of the extracellular domain, and anchoring adhesion to the actin or microtubule cytoskeleton through interaction with catenins.^99^ Differential regulation of cadherin expression plays a critical role in cell–cell interaction during NC cell EMT.^42^ The downregulation of E-cadherin, a type 1 cadherin associated with epithelial cell integrity, coincident with the upregulation of N-cadherin and cadherin-6b, initially defines the neuroepithelium and premigratory NC cells in zebrafish, mouse, and chick.^42^–^44^ Transitions in cadherin isoform expression also appear to be critical for cells to acquire a motile phenotype.^100^ The downregulation of N-cadherin and Cad6 occurs prior to NC cell migration, switching expression to a less adherent type 2 cadherin: Cad7 in chick and Cad11 in mouse and *Xenopus*.^10^,^42^,^46^,^47^ Conversely, continued expression of N-cadherin inhibits NC delamination by maintaining adherens junctions and sequestering *β*-catenin from functioning in cell signaling,^48^ highlighting the critical role of cadherin regulation in EMT. The involvement of cadherins in human cancer has also been well established. The disruption of junctional proteins, including E-cadherin, is required for cancer cell movement and thus is a prominent feature in most human carcinomas, leading to an invasive phenotype and poor prognosis.^45^ Deregulation of N-cadherin has also been implicated in cancer metastasis, promoting motility in human breast cancer cells regardless of their E-cadherin expression.^49^,^50^

Cellular polarity is established through the asymmetric distribution of cellular organelles and proteins, and is essential for a variety of cellular processes including directed cell migration. Disruption of cell polarity is one of the defining features of EMT in both NC cell development and tumor metastasis. Because they form initially as part of the neuroepithelium, NC cells exhibit an epithelial apical–basolateral polarity that is altered at the onset of EMT and migration. At the molecular level, apical–basolateral polarity is established by evolutionarily conserved polarity proteins that form multiprotein complexes. Polarity complexes are localized in specific cellular domains and maintain cellular polarity through mutually antagonistic interactions: the Crumbs complex [CRB(1-3)/Pals1/PatJ], localized at the apical cell cortex, stabilizes the localization of the Par complex (Par3/Par6/aPKC) at the tight junction. The Par complex is also excluded from the basolateral domain by the Scribble complex (Scribble/Dgl/Lgl).^51^ Although the processes of establishing and maintaining apical–basolateral cell polarity have been well studied, the mechanisms underlying the reorganization of cellular polarity during NC EMT remain unclear.

Nonetheless, despite the limited understanding of how polarity proteins are regulated during EMT, increasing evidence implicates disruption of polarity proteins in cancer progression and poor clinical prognosis. For example, the downregulation of Crumbs protein CRB3 is required for tumor formation in mouse epithelial cells and correlates with high levels of vimentin and reduced expression of E-cadherin, both hallmarks of EMT, suggesting that CRB3 may normally function to maintain tight junctions, apicobasal polarity, contact inhibited growth, and suppress migration and metastasis.^52^ Snail and Zeb1 have been shown to directly bind to the promoter and repress the transcription of the Crumbs gene, highlighting Crumbs regulation as a potential mediator of EMT.^53^–^55^ Par3 expression and subsequent stability of the PAR complex are regulated by transforming growth factor *β* (TGF*β*) signaling,^56^ a key regulator of EMT in tumor formation.^57^,^58^ This suggests that TGF*β* pathways may mediate loss of apicobasal cell polarity and drive EMT associated with cancer progression.^56^ In addition, the overexpression of aPKC, a component of the PAR complex, has also been observed in cancer progression and correlates with a poor clinical prognosis in ovarian cancer, breast cancer, and non-small cell lung cancer.^59^ The precise mechanisms by which polarity proteins function in NC EMT and in tumor progression remain unclear; however, accumulating evidence of their involvement highlights the potential importance of polarity proteins in both NC cell and cancer cell biology.

## NC CELLS AND METASTATIC CANCER CELLS USE SIMILAR MIGRATION STRATEGIES

Following delamination, NC cells migrate from the dorsal neural tube to disparate tissues where they will differentiate. Depending on the environment through which they transit, NC cells exhibit different cellular strategies during migration. Cranial NC cells, for example, migrate as sheets of cells, consisting of both leader and follower cells. The leading cells respond to chemoattractants, whereas the following cells utilize contact inhibition to maintain polarity and directionality in their migration to the ventral pharyngeal arches.^4^ Trunk and enteric NC cells exhibit a very different mechanism of migration whereby they move in streams or single-cell chains.^101^,^102^ Similarly, cancer cells can exhibit one or more of these strategies to migrate from the primary tumor, either in single cells or in sheets, typically along blood vessels to new tissues.^103^

There are several processes that are common between NC cell and cancer cell migration (Figure 3). First, the cells must break down the ECM through which they migrate using MMPs that degrade components of the ECM. MMP-2 and MMP-8 are expressed as NC cells exit the neural tube and begin migrating^60^,^61^ and are required for NC cell migration.^62^,^63^ Cancer cells also use MMPs for degradation of ECM during EMT and migration.^66^ As with NC cells, MMP-2 enhances cancer cell migration *in vitro*,^67^ in an orthotopic mouse model of breast cancer,^68^ and is associated with a decrease in disease-free survival in human prostate^69^ and non-small cell lung cancer patients.^70^ There is also evidence that MMP-2, along with MMP-9 and MMP-14, have a role in promoting invasion and angiogenesis in mice.^71^

ADAMs are another family of metalloproteinases involved in cell migration. In *Xenopus*, ADAM9 and ADAM13 are required for breakdown of the ECM by cranial NC cells^64^ and the cleavage of cadherin-11.^65^ Several of the ADAM family members have also been implicated in the progression of cancer through the inhibition of apoptosis and the promotion of cell proliferation and angiogenesis.^72^ Additional studies have shown a correlation between many of the ADAM proteins and high metastatic rate and poor prognosis in human patients,^73^–^76^ although the multiple signaling functions of ADAMs could also contribute to these results.

Once NC cell migration begins, both attractive and inhibitory signals from the environment guide their movement. A positive cue for NC migration is the chemokine SDF1/CXCL12 from the microenvironment, which activates the CXCR4 receptor within migrating NC cells. Expression of the SDF1 ligand in the pharyngeal arches, along with expression of the CXCR4 receptor in the anterior migratory stream of NC cells, is necessary for proper migration of the cranial NC to populate the craniofacial region in zebrafish^77^ and *Xenopus*.^78^ Similarly, SDF-1/CXCR4 signaling is also required in the mouse trunk in order for NC cells to migrate and populate dorsal root ganglia.^79^ The role of chemokines and their receptors in cancer cell biology and metastatic spread was first documented in metastatic human breast cancer.^80^ In the past decade, this signaling pathway has been implicated in the metastatic spread of a multitude of human tumors. The expression of chemokine receptors by tumor cells is thought to provide them with access to the normal migratory pathways utilized in development during organogenesis, as well as access to pathways that direct the migration of discrete populations of immune cells to specific target organs for the establishment of regional immunity.^81^ Furthermore, expression of chemokines by tumor cells has also been proposed to promote tumor cell growth, angiogenesis, and the formation of immunotolerant microenvironments.^81^

Directed migration of NC cells also relies on inhibitory signals to keep the migrating cells spatially organized. The key signals involved in this process are the ligands, ephrin and semaphorin. They are expressed in regions where the NC cells do not normally migrate, such as the scleratome in the trunk or the tissues in between streams of migrating cells.^8^,^82^ The receptors for these ligands, Eph and neuropilin, are expressed by cephalic NC cells. Activation of these signaling pathways prevents migration of NC cells into specific zones by inducing collapse of cellular projections.^8^ Loss of normal Eph/ephrin or neuropilin/semaphorin signaling results in ectopic migration of NC into other tissues and mixing of NC streams, ultimately preventing proper patterning of the embryo.^83^–^85^ There is evidence that guidance molecules including ephrin and semaphorin are misregulated in human cancers including lung and breast cancer, suggesting that these signals may have a role in tumor progression and metastasis; however, the complexity of these signaling interactions complicates our understanding of how these molecules function in a metastatic environment.^86^,^87^ Further understanding of the role of pathfinding in tumor cell invasion will likely yield a wealth of information on how tumor cells metastasize.

## CONCLUSIONS

NC cells and tumor cells both undergo dynamic processes to transition from an epithelial layer to migratory cells. The molecular interactions that govern these developmental processes in NC cells are echoed by the misregulation of these same processes throughout tumorigenesis and cancer cell migration, making NC EMT and migration an excellent model for understanding cancer formation, progression, and metastasis. Interestingly, the deregulation of several early developmental genes is necessary and/or sufficient for cancer progression, suggesting that these genes regulate key steps in cell proliferation, cell death avoidance, and invasiveness. Identifying how this reprogramming occurs, either within a stem-like cell residing in a mature tissue or within a mature fully differentiated cell, will be critical in raising our understanding of the progression of cancer. A vast array of molecular tools and model systems are available and have been used repeatedly in the study of the NC, which can provide great insight into the intricacies of human cancer. Many complex and intriguing questions still remain in the processes of EMT and migration, such as how cell adhesion molecules are regulated temporally and spatially, how migrating cells communicate with each other and the environment, how components of the ECM are regulated during migration, how cells at the end of migration begin to colonize within their target tissue, whether there is a mesenchymal-to-epithelial transition of migrating cells within the target tissue, and how this is regulated. Exploring these questions through the development of the NC may provide key insights into the regulation of tumor cell adhesion, communication, and migration through tissues during cancer metastasis, which will lead to the identification of disease predictors and potential therapeutic targets.
